# Antidepressant treatment initiation among children and adolescents with acute versus long COVID: a large retrospective cohort study

**DOI:** 10.1186/s13034-024-00787-z

**Published:** 2024-08-01

**Authors:** Phuong TM Tran, Alejandro Amill-Rosario, Susan dosReis

**Affiliations:** Department of Practice, Sciences, and Health Outcomes Research, University of Maryland School of Pharmacy, 220 Arch St, 12th Floor, Baltimore, MD 21201 USA

**Keywords:** Post-acute COVID-19 syndrome, Mental health, COVID-19, Antidepressant medications, Child and adolescent, Mood, Psychopharmacology

## Abstract

**Background:**

Child and adolescent antidepressant use increased post-pandemic, but it is unknown if this disproportionally affected those who develop post-acute sequelae of coronavirus disease 2019 (COVID) or long COVID. This study compared the risk of antidepressant initiation among children and adolescents with long COVID with those who had COVID but did not have evidence of long COVID.

**Methods:**

Our retrospective cohort study of children and adolescents aged 3–17 years at the first evidence of COVID or long COVID from October 1, 2021 through April 4, 2022 was conducted within Komodo’s Healthcare Map^™^ database. The index date was the earliest date of a medical claim associated with a COVID (COVID comparators) or long COVID diagnosis (long COVID cases). The baseline period was six months before the index date. The outcome was antidepressant initiation within twelve months after the index date. Due to the large number of COVID relative to long COVID cases, COVID comparators were randomly selected with a ratio of 2 COVID to 1 long COVID. We used propensity score matching to control for confounding due to imbalances in the baseline covariates. Log-binomial models estimated the relative risk (RR) of antidepressant initiation in the propensity score matched sample. We conducted several sensitivity analyses to test the robustness of our findings to several assumptions.

**Results:**

Our child and adolescent sample included 18 274 with COVID and 9137 with long COVID. Compared with those with COVID, a larger proportion of long COVID children and adolescents had psychiatric disorders, psychotropic use, medical comorbidities, were previously hospitalized, or visited the emergency department. In the propensity score-adjusted analysis, the long COVID group had a statistically significant higher risk of antidepressant initiation relative to the COVID comparator (adjusted-RR: 1.40, 95% CI = 1.20, 1.62). Our findings were robust across sensitivity analyses.

**Conclusions:**

The increased risk of antidepressant initiation following long COVID warrants further study to better understand the underlying reasons for this higher risk. Emerging evidence of long COVID’s impact on child mental health has important implications for prevention and early interventions.

**Supplementary Information:**

The online version contains supplementary material available at 10.1186/s13034-024-00787-z.

## Introduction

 The coronavirus disease (COVID) affected more than 651 million people worldwide, with an estimated 65 million struggling with post-acute sequelae, or long COVID [1]. Long COVID is a protracted illness lasting beyond 4–12 weeks following acute COVID infection [2]. Long COVID is an evolving condition that encompasses more than 200 complex and heterogeneous symptoms (i.e., fatigue, neurocognitive problems, mood disturbance, respiratory problems) that present beyond the acute COVID infection [1, 2]. The United States Centers for Disease Control and Prevention defines long COVID as ongoing symptoms lasting for three months or more, while the United Kingdom National Institute for Health and Care Excellence defines it as post-acute symptoms after four weeks [3]. In the absence of a standard, agreed-upon definition, [3] we considered all available evidence and adopted a definition as a protracted illness lasting 4–12 weeks after the initial acute COVID infection. The estimated global prevalence of long COVID varies widely from 4–66% [4]. The Centers for Disease Control and Prevention estimated that 1.3% of children and adolescents in the United States (US) reported ever having experienced long COVID in 2022 [5].

Long COVID may be associated with a greater burden of psychiatric conditions and a higher risk of developing mental health problems [6–8]. US adults with long COVID had a higher risk of mental health–related medical encounters for at least 6 months after COVID infection than before COVID infection [8]. An electronic health record study of 236 379 adults with long COVID showed that those with long COVID had a higher rate of psychiatric diagnoses (33.6%, of which 12.8% were first-time diagnoses) in the six months after the acute infection than those who had influenza or other respiratory tract infections [7]. Although adults with long COVID and COVID experience depression and anxiety symptoms, these symptoms are more common among those who had long COVID [6]. Most of the aforementioned studies have been in adults, thus there is a need for more research on the impacts of long COVID on children and adolescents.

Despite the low prevalence of long COVID in children and adolescents, it can have a debilitating effect, [2] as noted in the US President’s Memorandum on Addressing the Long-Term Effects of COVID-19 [9]. The COVID-19 pandemic had a profound impact on child and adolescent mental health [10]. Globally, depression, anxiety, and antidepressant use among children and adolescents increased during the COVID-19 pandemic and remained higher than pre-pandemic levels [11–16]. The increase was most notable among females and countries in North America and Europe [11, 13]. Far less is known about the impact of long COVID on child and adolescent mental health. While there is no effective treatment for long COVID, antidepressant (an important tool in the treatment of depression) might be useful in the management of psychiatric sequelae of long COVID [17]. Long COVID symptoms include depression, anxiety, and mood disorders, which can be managed by antidepressant treatment. In addition, emerging evidence shows that selective serotonin reuptake inhibitors can reduce severity and mortality of COVID infection, [18] although this has not been confirmed yet [19]. Antidepressant prescribing can provide complementary information regarding the burden and the receipt of care for psychiatric symptoms among children and adolescents with long COVID. This study aimed to examine antidepressant initiation following long COVID. We compared antidepressant initiation among children and adolescents aged 3–17 years old at first evidence of long COVID to those with COVID without evidence of long COVID. We hypothesized that children and adolescents with long COVID were at a higher risk of antidepressant initiation.

## Methods

### Study design and data sources

We conducted a retrospective cohort study using Komodo’s Healthcare Map™ database [20]. Komodo’s Healthcare Map™ is an administrative database of healthcare encounters, which is age and geographic representative of the US population with either privately or publicly funded health insurance​. In any given year, there are, on average, 200 million insured individuals across the age continuum, primarily employer-based insurance​ and a smaller proportion of publicly funded programs, and over 80 million with full-adjudicated medical and prescription claims. The study was approved by the Institutional Review Board of the University of Maryland Baltimore. We followed the Strengthening the Reporting of Observational Studies in Epidemiology (STROBE) reporting guidelines.

### Study population

**Fig. 1:**
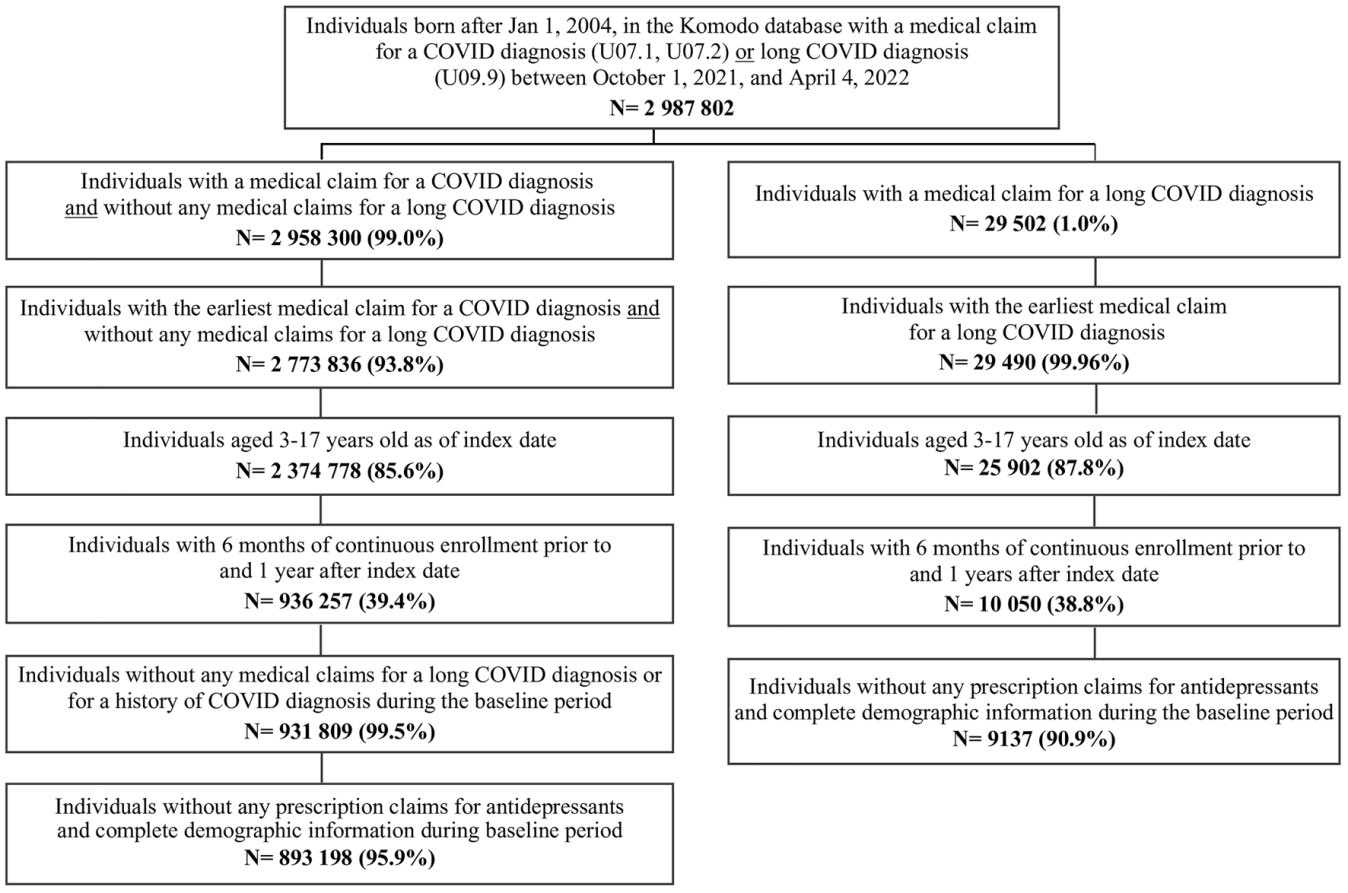
Attrition diagram and sample selection of the study cohort

The study population was US children and adolescents 3–17 years old. We limited the study cohort to those with the first evidence of a medical claim with a COVID or long COVID diagnosis on or after October 1, 2021, i.e., the date when the International Classification of Diseases, Tenth Revision, Clinical Modification (ICD-10-CM) code for long COVID (U09.9) became effective and thus identifiable in the data. We identified long COVID with this code, as this was used in clinical practice soon after its release to identify individuals with long COVID patients [21]. The index date for the start of follow-up was the earliest date of a medical claim associated with a long COVID or COVID diagnosis during the cohort accrual period from October 1, 2021, through April 4, 2022. The index date was required to be at least 12 months prior to the study end date (April 4, 2023) to allow for a 12-month follow-up for all subjects. We required continuous insurance enrollment six months before the index date (i.e., baseline period) and 12 months after the index date (i.e., outcome assessment period). We excluded those with missing information for year of birth and sex or with any antidepressant prescription claims in the six-month baseline period. For the COVID group, we also excluded children and adolescents with diagnosis codes of a long COVID or history of COVID (ICD-10-CM code Z86.16) within the six-month baseline period (Fig. 1).

### Exposure

Exposure was the first evidence of COVID and long COVID status. Children and adolescents were designated to the long COVID cohort if they had at least one medical claim associated with ICD-10-CM code U09.9 in the cohort accrual period. We designated children and adolescents as COVID if they had at least one medical claim associated with an ICD-10-CM code U07.1 or U07.2. The COVID group could not have any medical claims with a long COVID diagnosis during the baseline or cohort accrual period.

We did not include the ‘no-exposure’ group, i.e., those who did not have claims of a COVID and long COVID diagnosis, as this would dilute the study’s focus from the impact of the COVID-19 infection to the more general impact of the pandemic. The effect of the quarantine measures on youth’s mental health are largely unmeasurable in any data source. It is known that the COVID-19 pandemic affected physical activities, access to entertainment, positive familial relationships, and social support due to different social restriction measures, leading to worsened mental health outcomes in adolescents [22, 23]. Additionally, it is potential that there are undetected COVID and long COVID cases in our data. We considered these issues and felt that it would introduce bias that we would not be able to adjust for in the analytic models. On the other hand, the COVID and long COVID cohorts were nested in a population that was exposed to the quarantine measures of the pandemic and the COVID infection. Thus, by comparing long COVID with COVID cohorts, we could isolate the effect of the COVID infection, since all other factors would be held equal.

### Outcome

The study outcome was antidepressant initiation, defined as having at least one pharmacy claim for antidepressant medication within 12 months after the index date. Antidepressant medications included selective serotonin reuptake inhibitors, serotonin norepinephrine reuptake inhibitors, tricyclic antidepressants, and other antidepressants (Additional File 1 Table S1).

### Covariates

Covariates were assessed in the six-month baseline period. These included age, sex, and other mental health diagnoses (i.e., anxiety, depression, bipolar disorder, adjustment disorder, attention-deficit/hyperactivity disorder (ADHD), seizure, and schizophrenia), psychotropic medications (i.e., anxiolytics, antipsychotics, mood stabilizers, sedatives, and ADHD medications), medical conditions (i.e., cancer, diabetes, immunocompromised conditions, gastrointestinal conditions and respiratory conditions), and healthcare utilization (i.e., hospitalization and emergency department visits). Additional File 1 Table S1 lists the ICD-10 codes and generic drug names used to identify baseline covariates.

### Statistical analysis plan

Due to the large number of COVID relative to long COVID cases, we randomly selected two COVID cases for every long COVID case to create the analytic comparator cohort. Descriptive measures of baseline demographics and clinical covariates assessed differences between long COVID and COVID groups. We used propensity score (PS) methods to control for confounding due to imbalances in the baseline covariates. Logistic regression estimated the PS as the probability of long COVID status given the measured baseline covariates. We implemented PS using a 1-to-1 greedy matching without replacement and a 0.15 caliper [24]. The absolute standardized difference (ASD) evaluated covariate balance before and after PS matching, with an ASD < 0.1 as evidence of good balance. In the PS-matched sample, log-binomial models estimated the risk ratio of antidepressant initiation among long COVID relative to COVID groups.

Sensitivity analyses tested the robustness of our findings to several assumptions. To address potential misclassification of long COVID cases, we excluded those with COVID who developed long COVID in the 12-month outcome assessment period. To minimize the potential inclusion of prevalent long COVID cases, we excluded those who had unofficial long COVID diagnosis codes (i.e., suggested codes before official COVID-19 ICD-10 codes were available) before October 1, 2021. To account for the COVID infection waves associated with different variants, we included the month/year of diagnosis date in our outcome model. Finally, since US states differed in the COVID prevalence and pandemic response, [25] we generated robust standard errors, using a multilevel log-binomial model, clustered at the state level. We determined statistical significance using the 95% confidence interval (CI) or *p* < 0.05 (2-sided). All analyses were performed using SAS^®^ version 9.4.

## Results

### Characteristics of the study cohort

We identified 893 198 COVID and 9137 long COVID children and adolescents from October 1, 2021 through April 4, 2022 (Fig. 1). Before PS matching, the analytic sample of two COVID for every long COVID case included 18 274 COVID and 9137 long COVID children and adolescents. Compared with the COVID group, a larger proportion of the long COVID group were aged 14–17 years old (40.5% versus 26.2%), had immunocompromised conditions (1.1% versus 0.5%), respiratory conditions (17.7% versus 9.3%), and gastrointestinal conditions (19.0% versus 10.1%), and had emergency department visits (19.0% vs. 13.0%) and hospitalization encounters (5.4% vs. 0.8%) in the six-month baseline period (Table 1). There were statistically significant differences between the long COVID and COVID groups, respectively, in terms of depression (2.6% versus 1.4%), anxiety (7.2% versus 3.1%), and adjustment disorder (4.8% versus 2.9%), and psychotropic medication use other than antidepressants, such as anxiolytics (2.0% versus 1.3%), mood stabilizers (0.6% versus 0.4%), sedatives (0.3% versus 0.1%) and ADHD medications (5.6% versus 4.7%).

**Table 1:** Baseline characteristics of COVID and long COVID children and adolescents before and after PS matching

	Before matching				After matching		
	COVID (*n* = 18 274) Frequency (%)	Long COVID (*n* = 9137) Frequency (%)	ASD^a^	*p* value^b^	COVID (*n* = 8443) Frequency (%)	Long COVID (*n* = 8443) Frequency (%)	ASD^a^
**Demographics at baseline**							
*Age as of index date* ^c^							
3–4 years old	2196 (12.0)	668 (7.3)	**0.32**	**< 0.001**	647 (7.7)	660 (7.8)	0.02
5–9 years old	5926 (32.4)	2155 (23.6)			2043 (24.2)	2043 (24.2)	
10–13 years old	5357 (29.3)	2613 (28.6)			2480 (29.4)	2495 (29.6)	
14–17 years old	4795 (26.2)	3701 (40.5)			3273 (38.8)	3245 (38.4)	
*Sex*							
Female	8908 (48.8)	4491 (49.2)	**0.16**	0.53	4143 (49.1)	4059 (48.1)	0.01
Male	9366 (51.3)	4646 (50.9)			4300 (50.9)	4384 (51.9)	
**Psychiatric disorders at baseline**							
Depression	256 (1.4)	241 (2.6)	0.09	**< 0.001**	188 (2.2)	213 (2.5)	0.02
Anxiety	569 (3.1)	660 (7.2)	**0.19**	**< 0.001**	497 (5.9)	410 (4.9)	0.05
ADHD^d^	955 (5.2)	589 (6.5)	0.05	**< 0.001**	550 (6.5)	540 (6.4)	0.01
Adjustment disorder	530 (2.9)	434 (4.8)	**0.10**	**< 0.001**	365 (4.3)	380 (4.5)	0.01
Bipolar disorder	28 (0.2)	18 (0.2)	0.01	0.40	15 (0.2)	18 (0.2)	0.01
Seizure	204 (1.1)	184 (2.0)	0.07	**< 0.001**	132 (1.6)	136 (1.6)	0.00
Schizophrenia	17 (0.1)	10 (0.1)	0.01	0.68	< 10	< 10	0.00
**Psychotropic use at baseline**							
Anxiolytics	240 (1.3)	179 (2.0)	0.05	**< 0.001**	137 (1.6)	157 (1.9)	0.02
Antipsychotics	102 (0.6)	50 (0.6)	0.00	0.91	42 (0.5)	48 (0.6)	0.01
Mood stabilizers	65 (0.4)	51 (0.6)	0.03	**0.01**	45 (0.5)	47 (0.6)	0.00
Sedatives	22 (0.1)	26 (0.3)	0.04	**0.002**	17 (0.2)	21 (0.3)	0.01
ADHD medications	855 (4.7)	515 (5.6)	0.04	**< 0.001**	480 (5.7)	474 (5.6)	0.00
**Medical comorbidities at baseline**							
Cancer	44 (0.2)	30 (0.3)	0.02	0.19	23 (0.3)	28 (0.3)	0.01
Diabetes	99 (0.5)	60 (0.7)	0.01	0.24	53 (0.6)	53 (0.6)	0.00
Immunocompromised conditions	91 (0.5)	100 (1.1)	0.07	**< 0.001**	65 (0.8)	78 (0.9)	0.02
Respiratory conditions	1690 (9.3)	1617 (17.7)	**0.25**	**< 0.001**	1324 (15.7)	1440 (17.1)	0.04
Gastrointestinal conditions	1837 (10.1)	1734 (19.0)	**0.26**	**< 0.001**	1312 (15.5)	1336 (15.8)	0.01
**Healthcare utilization at baseline**							
Any emergency department visits	2382 (13.0)	1735 (19.0)	**0.16**	**< 0.001**	1348 (16.0)	1350 (16.0)	0.00
Any hospitalizations	151 (0.8)	489 (5.4)	**0.26**	**< 0.001**	151 (1.8)	167 (2.0)	0.01

^a^Absolute standardized difference, in bold ASD > 0.1; ^b^p-value from a chi-square test, in bold p-value < 0.05

^c^Percentages may not add up to 100% due to rounding; ^d^Attention-deficit/hyperactivity disorder

After PS matching, the sample had 8443 children and adolescents in each group. The baseline covariates were well-balanced, as evidenced by an ASD < 0.10 (Table 1). Characteristics of those excluded from the PS-matched COVID and long COVID groups are in Additional File 1 Table S2.

### Antidepressant initiation

The distributions of antidepressant therapeutic subclass initiation among COVID-19 and long COVID children and adolescents are in Table 2, with selective serotonin reuptake inhibitors being the most common antidepressant in both groups.

**Table 2:** Antidepressant therapeutic subclass initiation among COVID-19 and long COVID children and adolescents

Antidepressant drug class	COVID-19 *n* (%)	Long COVID *n* (%)
Selective serotonin reuptake inhibitors	357 (2.0)	314 (3.4)
Serotonin and norepinephrine reuptake inhibitors	< 10	15 (0.2)
Tricyclic antidepressants	23 (0.1)	102 (1.1)
Other antidepressants	45 (0.3)	33 (0.4)
Total in each cohort	18 274	9137

The risk ratios for antidepressant initiation, before and after PS matching, are in Table 3. After PS matching, 3.4% (283/8443) of COVID and 4.7% (395/8443) of long COVID children and adolescents initiated an antidepressant. In the PS-matched analysis, the long COVID group had statistically significant higher risks of initiating antidepressants (RR = 1.40, 95% CI = 1.20, 1.62, *p* < 0.0001) relative to the COVID group (Table 3).

**Table 3:** Risk of antidepressant initiation among long COVID compared with COVID children and adolescents

Exposure​	Total​	Antidepressant initiation (*n*, %)​	RR (95%CI)​	*p* value
Before PS matching				
COVID​	18 274	434 (2.4)	Ref​	
Long COVID​	9137	464 (5.1)	**2.20 (1.93–2.51)**	**< 0.001**
After PS matching				
COVID​	8443	283 (3.4)	Ref​	
Long COVID​	8443	395 (4.7)	**1.40 (1.20–1.62)**	**< 0.001**

In bold, p < 0.05

All sensitivity analyses produced risk estimates consistent with the primary analysis (Table 4).

**Table 4:** Sensitivity analyses of the primary analysis of the risk of antidepressant initiation

Sensitivity analyses​	RR (95%CI)​	*p* value
1. Excluded COVID cases who developed long COVID during following-up time​	1.64 (1.41–1.91)	< 0.001
2. Adjusted for the month of diagnosis in the outcome model​	1.42 (1.23–1.66)	< 0.001
3. Adjusted for the clustering effect of states​	1.51 (1.32–1.72)	< 0.001
4. Excluded long COVID cases who had medical claims with unofficial long COVID codes during the baseline period​	1.43 (1.23–1.66)	< 0.001

## Discussion

In this cohort study of US children and adolescents aged 3–17, we found that long COVID was associated with a higher risk of antidepressant initiation relative to COVID. Relative to the COVID group, a higher proportion of the long COVID group in this study had medical and mental health conditions and had used healthcare services. This suggests a higher burden of baseline illness among children and adolescents in the long COVID group compared with the COVID group.

The higher risk of antidepressant initiation among children and adolescents with long COVID was observed even after adjusting for imbalances in baseline medical and mental health disorders. Previous studies report that individuals with long COVID have a greater burden of psychiatric conditions [6, 26]. Research shows that children and adolescents with long COVID experience symptoms, including mood (17%), fatigue (10%), sleep disorders (8%), and headache (8%) [2]. The progression of pre-existing psychiatric conditions and chronic conditions among long COVID individuals might also contribute to the higher risk of antidepressant treatment initiation. A study using electronic health records from nine US children’s hospitals showed that, compared to children and adolescents with COVID who did not develop long COVID, those with long COVID were more likely to be hospitalized during the acute COVID infection phase or have complex chronic conditions [27]. A systematic review in adult populations suggests that comorbid anxiety and depression are associated with increased risk of long COVID, [28] but there is very little research on long COVID in children and adolescents. Research in adults with long COVID shows increased social stigma, mental health stress, and medical misdiagnosis [29, 30]. Adults with long COVID have higher rates of depression, anxiety, and suicidality compared to those with COVID [6]. Their rates of depression, anxiety, and suicidality are similar to individuals with chronic and debilitating conditions, such as cancer or diabetes [6]. A new study suggests that serotonin reduction among long COVID individuals may explain the mechanism of psychiatric symptoms, [31] which may also explain the increased risk of antidepressant treatment initiation. Extrapolating from adults, it is possible that our finding of a higher risk of antidepressant initiation among those with long COVID may be due to a greater psychiatric burden subsequent to long COVID.

Emerging evidence suggests a protective effect of antidepressants against COVID infection [18, 32, 33]. This may encourage providers to initiate antidepressant medication. Because our study could not ascertain the indications for antidepressant initiation, the underlying factors that drive antidepressant initiation among children and adolescents with long COVID are unclear. Studies on the increased risk of antidepressant treatment among children and adolescents with long COVID, especially those exploring the indications for antidepressant initiation, are warranted.

This study has several strengths. This study leveraged the Komodo’s Healthcare Map,™ which is a large database representative of the US. The database contained more than 1 million children and adolescents with COVID, enabling a large sample for this study. Our study minimized prevalent user bias by restricting the cohort to new evidence of COVID. We used an active comparator cohort, comparing long COVID with COVID, in order to minimize potential healthy user bias. Despite these strengths, we acknowledge several limitations of our study. First, long COVID symptoms are difficult to distinguish from symptoms of depression, such as anxiety, mood disorders, or chronic fatigue. This could have led to underreporting of long COVID. Our sensitivity analysis attempted to adjust for misclassification. Second, we were only able to detect COVID and long COVID cases that received medical attention, and it is possible that there were many un-detected cases in the data. To mitigate this potential misclassification bias owing to un-detectable cases in the data, we used an active comparator design rather than an un-infected comparator. Third, the use of the ICD-10-CM U09.9 code to identify long COVID may have led to the underrepresentation of males, Hispanic ethnicity, and people of low socioeconomic status. Prior studies report that the U09.9 code was most commonly used for females, Whites, and people living in areas with low poverty and low unemployment [34]. Fourth, it was not possible to identify the timing of COVID infection in relation to the development of long COVID, especially with the lack of a standard definition of long COVID. Finally, our data did not have information on the severity of the COVID infection, vaccination status, race/ethnicity or socioeconomic status, and thus, we were unable to evaluate potential confounding by these factors. Our population primarily had private commercial insurance, which limited generalizability to those with public insurance or the uninsured. Lower socioeconomic status is associated with higher levels of psychiatric problems in those with long COVID, [6] thus our study may have under-represented antidepressant initiation.

 Long COVID is likely to have a substantial and multi-faceted public health impact [35]. Emerging evidence reveals new symptoms of long COVID and its debilitating impact on physical and mental health [1, 6, 27, 35]. Long COVID is now recognized as a disability under Title II (state and local government) and III (public accommodations) of the Americans with Disabilities Act, Sect. 504 of the Rehabilitation Act of 1973, and Sect. 1557 of the Patient Protection and Affordable Care Act [36]. Despite the burden of mental health issues among children and adolescents with long COVID, four years after the COVID-19 pandemic, the underlying biological mechanism, the complete list of symptoms, and the effective treatment of long COVID remain unresolved. There is growing US federal investment in research to tackle the long-term consequences of COVID infection [9, 37]. The National Institutes for Health has invested more than $1.5 billion into the Researching COVID to Enhance Recovery Initiative [37]. Our study is the first, to our knowledge, to report on an increased risk of antidepressant initiation among children and adolescents with long COVID. This contributes to the existing literature on the impact of long COVID on child and adolescent health. Our findings inform the need for treatment and management of mental health and well-being of children and adolescents with long COVID. This raises awareness of the mental health needs of children and adolescents and their caregivers, including access to healthcare for screening and treatment of mental health conditions. For those most affected and requiring antidepressant treatment, ongoing monitoring should be in place. With the growing burden of long COVID coupled with the long-term negative effect of COVID-19 pandemic on the development and mental health of children, [38] it is critical to prioritize the mental health and well-being of children and adolescents as this will have impacts for generations to come.

## Conclusion

Our study found that children and adolescents with long COVID had a higher risk of antidepressant initiation relative to those with COVID. The results highlight a need for symptom monitoring and mental health management among children and adolescents who develop long COVID. Future studies evaluating the changes in treatment prescriptions and dosages among youth initiating or using antidepressants and their potential effectiveness in reducing severity and psychiatric symptoms for long COVID patients are needed. A better understanding of the manifestation and impact of long COVID in the pediatric population could better inform approaches to address their mental health and well-being.

### Electronic supplementary material

Supplementary Material 1

## Data Availability

Not applicable. Individual-level data cannot be made available due to confidentiality. Data use agreements with the vendor do not permit the release of these data. For further information on data access, please contact Komodo’s Healthcare Map.

## Abbreviations

ADHD: Attention-deficit/hyperactivity disorder
COVID: Coronavirus disease 2019
Long COVID: Post-acute sequelae of coronavirus disease 2019
RR: Relative Risk
US: United States
