# Supplementary material for: Antidepressant treatment initiation among children and adolescents with acute versus long COVID: a large retrospective cohort study
Source: Child Adolesc Psychiatry Ment Health. 2024 Aug 1;18:95. doi: 10.1186/s13034-024-00787-z (PMC11295664; doi:10.1186/s13034-024-00787-z)
Supplement: Supplementary file 1 — Supplementary Material 1 [file 13034_2024_787_MOESM1_ESM.doc]

**Table S1: International Classification of Diseases, Tenth Revision, Clinical Modification (ICD-10-CM) codes for comorbid mental health disorders and medical conditions and generic drug names for medication therapeutic classes.**

| **Feature** | **Generic drug names/ ICD-10-CM diagnosis code** |
| --- | --- |
| **Outcome** |  |
| Antidepressants | **Selective serotonin reuptake inhibitors (SSRIs):** citalopram, fluoxetine, fluvoxamine, escitalopram, sertraline, paroxetine.  **Serotonin norepinephrine reuptake inhibitors (SNRIs)**: desvenlafaxine, venlafaxine, duloxetine, levomilnacipran, milnacipran,  **Tricyclic antidepressants (TCAs):** imipramine, clomipramine, trimipramine, amitriptyline, protriptyline, nortriptyline, doxepin, amoxapine, desipramine.  **Other antidepressants**: bupropion,  mirtazapine, nefazodone, phenelzine, trazodone, vortioxetine, and vilazodone |
| **Mental health conditions** |  |
| Depression | F32, F33 |
| Anxiety | F40, F41 |
| ADHD | F90 |
| Adjust disorder | F43, F43.0, F94.1, F94.2 |
| Bipolar disorder | F31 |
| Seizure | G40, G41, R56 |
| Schizophrenia | F20, F21, F22, F23, F24, F25, F28, F29 |
| **Comorbid conditions** | |
| Cancer | C0-C8, C91-C97 |
| Respiratory conditions (chronic obstructive pulmonary disease, other chronic lower respiratory disease, asthma) | J40-47, excluding J45 |
| Diabetes | E10-E14 |
| Immunocompromised (HIV, hematologic malignancy, immune-mediated inflammatory disease, solid organ) | B20-B24, C8, C91-C96  M02.3, M05, M06, M07, M08, M31.0, M31.3, M31.6, M31.7, M31.8, M32, M33, M34, M35.0, M35.1, M35.2, M35.3, M35.4, M35.9, M45, K50, K51, L10, L40 |
| Gastrointestinal conditions | K21.0, K21.9, K27, K30, K50, K51, K57, K58, K59.04, R11 |
| Unofficial long COVID codes used at CDC’s initial recommendation before the official code’s release | Code U07.1, followed by B94.8  Source: Coronavirus disease (COVID-19): Post COVID-19 condition. https://www.who.int/news-room/questions-and-answers/item/coronavirus-disease-(covid-19)-post-covid-19-condition. |
| **Psychotropic use** | |
| Anxiolytics | Chlordiazepoxide, diazepam, hydroxyzine, clonazepam, ozazepam, meprobamate, alprazolam, buspirone, clorazepam, clonazepam, halazepam, prazepam |
| Antipsychotics | First generation: chlorpromazine, fluphenazine, haloperidol, loxapine, perphenazine, pimozide, and thioridazine  Second generation: aripiprazole, asenapine, brexpiprazole, cariprazine, clozapine, iloperidone, lurasidone, lumateperone, olanzapine, olanzapine, olanzapine/fluoxetine, paliperidone, pimavanserin, quetiapine, risperidone, and ziprasidone |
| Mood stabilizers | Carbamazepine, divalproex sodium, gabapentin, lamotrigine, levetiracetam, lithium, oxcarbazepine, topiramate, and valproic acid |
| Sedatives | Amobarbital, chloral hydrate, flurazepam, methohexital, midazolam, pentobarbital, tasimelteon, butabarbital, estazolam, eszopiclone, ethchlorvynol, glutethimide, lemborexant, phenobarbital, quazepam, ramelteon, suvorexant, temazepam, triazolam, zaleplon, zolpidem |
| ADHD Medications | Stimulants: Amphetamine, Amphetamine/Dextroamphetamine, Dextroamphetamine, Dexmethylphenidate, Lisdexamfetamine, Methylphenidate, Methamphetamine, And Pemoline  Non-Stimulants: Atomoxetine, Clonidine, And Guanfacine |

**Table S2: Characteristics of long COVID and COVID children and adolescents excluded from the analytic** sample

|  | **COVID (n= 9831)** | **Long COVID (n= 694)** |  |
| --- | --- | --- | --- |
|  | **Frequency (%)** | **Frequency (%)** |  |
|  |
| **Demographics at baseline** | | |  |
| **Age as of index date^a^** | | |  |
| 3-4 years old | 1549 (15.8) | <10 |  |
| 5-9 years old | 3883 (39.5) | 112 (16.1) |  |
| 10-13 years old | 2877 (29.3) | 118 (17.0) |  |
| 14-17 years old | 1522 (15.5) | 456 (65.7) |  |
| **Sex^a^** | | |  |
| Female | 4765 (48.5) | 432 (62.3) |  |
| Male | 5066 (51.5) | 262 (37.8) |  |
| **Psychiatric disorders at baseline** | | |  |
| Depression | 68 (0.7) | 28 (4.0) |  |
| Anxiety | 72 (0.7) | 250 (36.0) |  |
| ADHD^b^ | 405 (4.1) | 49 (7.1) |  |
| Adjustment disorders | 165 (1.7) | 54 (7.8) |  |
| Bipolar disorder | 13 (0.1) | <10 |  |
| Seizure | 72 (0.7) | 48 (6.9) |  |
| Schizophrenia | 11 (0.1) | <10 |  |
| **Psychotropic use at baseline** | | |  |
| Anxiolytics | 103 (1.1) | 22 (3.2) |  |
| Antipsychotics | 60 (0.1) | <10 |  |
| Mood stabilizers | 20 (0.2) | <10 |  |
| Sedatives | <10 | <10 |  |
| ADHD medications | 375 (3.8) | 41 (5.9) |  |
| **Medical comorbidities at baseline** | | |  |
| Cancer | 21 (0.2) | <10 |  |
| Diabetes | 46 (0.5) | <10 |  |
| Immunocompromised conditions | 26 (0.3) | 22 (3.2) |  |
| Respiratory conditions | 366 (3.7) | 177 (25.5) |  |
| Gastrointestinal conditions | 525 (5.3) | 398 (57.4) |  |
| **Healthcare utilization at baseline** | | |  |
| Any emergency department visits | 1034 (10.5) | 385 (55.5) |  |
| Any hospitalizations | 0 (0.0) | 322 (46.4) |  |

| ^a^Percentages may not add up to 100% due to rounding; ^b^Attention-deficit/hyperactivity disorder |
| --- |
|  |
